# Method for improving the quality of genomic DNA obtained from minute quantities of tissue and blood samples using Chelex 100 resin

**DOI:** 10.1186/s12575-018-0077-6

**Published:** 2018-06-01

**Authors:** Utkarsha A. Singh, Mukta Kumari, Soumya Iyengar

**Affiliations:** 1 Division of Systems Neuroscience, National Brain Research Centre, Deemed University, Gurugram, Haryana India; 2 Professor, Systems Neuroscience, National Brain Research Centre, NH-8, Manesar, Gurgaon, Haryana 122051 India

**Keywords:** DNA Isolation, Genomic DNA, Chelex 100, Zebra finches, House crows

## Abstract

**Background:**

Although genomic DNA isolation using the Chelex 100 resin is rapid and inexpensive, the DNA obtained by this method has a low concentration in solution and contains suspended impurities. The presence of debris in the DNA solution may result in degradation of DNA on long term storage and inhibition of the polymerase chain reaction. In order to remove impurities and concentrate the DNA in solution, we have introduced modifications in the existing DNA isolation protocol using Chelex-100. We used ammonium acetate to precipitate proteins and a sodium acetate- isopropanol mixture to pellet out DNA which was washed with ethanol.

**Results:**

A pure DNA pellet that can be dissolved in water or Tris-EDTA buffer and stored for a long time at − 80 °C was obtained. We also observed a 20-fold change in the DNA concentration following precipitation and re-dissolution.

**Conclusion:**

Our method is different from other extraction methods since it uses non-toxic, easily available and inexpensive reagents as well as minimal amounts of blood or tissue samples for the DNA extraction process. Besides its use in sex determination and genotyping in lab animals as described in this paper, it may also have applications in forensic science and diagnostics such as the easy detection of pathogenic DNA in blood.

## Background

Genomic DNA finds diverse applications in the study of mutations, genome structures, DNA fingerprinting and in the creation of genomic libraries. The varied usage of genomic DNA has brought multiple DNA extraction methods into existence. Some of the widely used DNA extraction methods include chloroform-based extraction [1], silica-based extraction (QIAmp DNA mini kit, Qiagen), [2] and magnetic separation [3–5]. Whereas chloroform-based DNA extraction requires the use of toxic chemicals, magnetic separation and silica-based DNA extraction tend to be expensive.

A major drawback, common to all these methods, is the requirement of large quantities of biological samples for DNA extraction. To obtain ample amounts of genetic material for PCR reactions from trace quantities of biological samples, Singer-Sam [6] suggested the use of an ion-exchanging resin (Chelex 100) for the DNA extraction process. Chelex 100 (Bio-Rad Laboratories, CA, USA) is a styrene-divinylbenzene copolymer containing paired iminodiacetate ions. It acts by chelating transition metal ions, the selectivity of which depends upon iminodiacetic acid. The cation exchanging ability of the resin is functional at neutral or weakly acidic pH (> 4.0). At very low pH, the resin begins to function as an anion exchanger. Hence, Chelex is categorized as a weakly acidic cation exchanger with high affinity for divalent metal ions.

The first protocol for DNA extraction using Chelex 100 was developed by Walsh et al. [7]. This method, which has found application mostly in forensics, involves heat denaturation of cells which may be attached to paper or fabric, in a solution containing Chelex 100 resin. High temperatures result in the release of DNA into the solution as well as facilitate the binding of Chelex resin to magnesium ions. Magnesium ions serve as cofactors to deoxyribonucleases and aid in their activation. Since magnesium ions are rendered unavailable to bind to deoxyribonucleases, DNA degradation is averted. After this protocol was established, the Chelex 100 resin became the method of choice for protocols requiring the rapid extraction of DNA from trace amounts of biological samples.

We needed to determine the sex of zebra finches (*Taeniopygia guttata)* and house crows (*Corvus splendens*), for some of the experiments in our lab. In order to perform PCR reactions for avian sex chromosomes Z- and W- [8, 9], we isolated genomic DNA from blood and tissue samples of these birds. However, even after multiple iterations of the standard Chelex protocol [7, 9], we found that on an average, the 260/230 ratio for DNA was 0.4 and the concentration of DNA was 40 ng/μl. We also found that the DNA extract was impure and pigmented due to suspended cellular debris.

Earlier studies have shown that organic debris in the form of proteins, for example, haemoglobin in blood and other compounds such as lactoferrin, IgG, and myoglobin are known to inhibit polymerase activity during PCR reactions [10]. Long term storage of impure DNA samples may also lead to the binding of these compounds to the DNA double helix, which may result in the inhibition of the PCR reaction. To purify and concentrate the DNA further, we introduced modifications in the existing Chelex protocol that involved removal of proteins and cellular debris as well as precipitation, washing and resuspension of DNA. When compared to results obtained using the protocol described by Soderstrom [9], our modified Chelex-based method yielded a 6.3-fold increase in quality (260 and 230 nm ratio of 2.35 compared to a median value of 0.375 using the old protocol). Our method also yielded an approximately 20-fold increase in the quantity of DNA (275.25 ng/μl versus a median value of 13.2 ng/μl using the earlier protocol) for the same amount of the sample. In the present study, we report in detail a method of isolation of genomic DNA from liver tissue samples and the quality and quantity of DNA thus obtained, using the Chelex resin. Additionally, we have also tried to quantify the minimum amount of tissue samples required to obtain a good yield of genomic DNA.

A major drawback of DNA extraction using Chelex resin is the contaminated DNA extract obtained at the end of the procedure. The method that we present here ensures the purification of the extract and minimal loss of the quantity of DNA from minute samples. Hence, along with specifying the quality and quantity, this protocol also ensures the purity of DNA obtained without the use of toxic or expensive chemicals. The DNA obtained by our protocol may find application in the detection of single nucleotide polymorphisms (SNPs), which may further be utilised for genotypic screening and also in the targeted sequencing of specific genes in order to study their role in a particular phenotype.

## Methods

### Reagents

Chelex 100 Resin (BioRad, Cat no #142–1253), Tris Buffer GR (Merck, 61,771,405,001,730), EDTA (Sigma-Aldrich, E9884-500G), Ammonium acetate (Merck, 61,750,105,001,046), Sodium acetate (Qualigens, 15,955), Isopropanol (Sigma-Aldrich, I9516-500ML), Ethanol (Merck, K45420483412).

### Buffers

1 M Tris solution (pH 8.0), 0.5 M EDTA (pH 8.0), 10X TE (Tris EDTA) Buffer (pH 8.0), 5% Chelex suspension (pH 8.0, to be prepared in 1X TE buffer), 7.5 M Ammonium acetate, 3 M Sodium acetate, 10 N NaOH (for pH adjustment, to be made in autoclaved deionized water), 50X Tris Acetate EDTA buffer (TAE buffer, pH 8.6), 5× Tris Borate EDTA buffer (TBE buffer pH 8.3), 30% Acrylamide Bis Acrylamide solution (Table 1).

**Table 1:** Recipes for Buffers used for the Protocol

Components	Volume
50X TAE Buffer (500 ml)	
Tris Buffer GR	121 g
EDTA Stock (0.5 M)	50 ml
Glacial Acetic Acid	28.55 ml
5× TBE Buffer (1 l, pH 8.3)	
Tris Buffer GR	54 g
Boric Acid	27.5 g
EDTA Stock (0.5 M)	20 ml
30% Acrylamide (25 ml)	
Acrylamide	7.3 g
Bis-acrylamide	0.2 g
8% Polyacrylamide Gel	
30% Acrylamide	2.620 ml
5x TBE Buffer	1.97 ml
Autoclaved Milli Q water	5.24 ml
10% Ammonium Persulfate	163 μl
TEMED	8 μl
Total	10 ml
EtBr (for staining)	0.5 μg/ml in 1X TBE
10X TE Buffer (pH 8.0)	
Tris Buffer	1 ml (Stock 1 M; Final concentration 100 mM)
EDTA	200 μl (Stock 1 M; Final concentration 10 mM)
Autoclaved Milli Q water	8.8 ml

### Blood and tissue sample preparation

All experiments conducted on birds (zebra finches, *n* = 5; Indian house crows, *n* = 5) as well as trans-cardiac perfusion with fixatives at the end of terminal experiments following an overdose of the anaesthetic ketamine were approved by the Institutional Animal Ethics Committee at the National Brain Research Centre, Manesar, which are in accordance with regulations provided by the Committee for the Purpose of Control and Supervision of Experiments on Animals, India. Approximately 5 μl of blood was adsorbed onto a disc of Whatman filter paper (No. 1001917) created by using a sterile paper punch (radius, 4 mm; thickness, 180 μm). The discs were then left to dry in a hood equipped with laminar flow for 30 min. In order to assess the smallest quantity of blood or tissue sample that was required to obtain an ample DNA yield for a PCR reaction, we used two, one, half and one-fourth of a Whatman filter paper disc and subjected them to our modified Chelex 100 DNA extraction protocol. The discs were separated to prevent them from sticking to each other after drying. For tissue, samples weighing 7 mg, 9 mg and 11 mg were excised from crow liver and slightly macerated with a pair of forceps, after which they were placed in Eppendorf tubes for further processing.

### Chelex Isolation protocol

Chelex solution (200 μl of 5% stock) was added to 1.5 ml Eppendorf tubes. These tubes were heated at 100 °C for 10 min in a boiling water bath. Whatman paper discs with blood samples or the liver tissue samples were then added to the hot Chelex solution. The Chelex suspension along with the paper disc or tissue samples was further heated for 8 min, vortexed and again heated for 7 min. The Eppendorf tubes were centrifuged at 12000 g for one and a half minutes at room temperature. The supernatant was pipetted out gently to avoid extracting the Chelex resin as well. The yield and quality of DNA in each sample of the supernatant was measured using a spectrophotometer [Nanodrop 1000 (ND 1000 V3.8.1, Thermo Fisher)].

### Protein precipitation

A 7.5 M stock solution of Ammonium Acetate was added to the supernatant collected after Chelex extraction, so that the working concentration of ammonium acetate in solution was 2.5 M [11, 12]. A thick yellowish-white precipitate of protein appeared immediately after adding ammonium acetate. This solution was allowed to rest for 5 min on ice. The sample was vortexed for 5 s and protein was pelleted out by centrifugation at 12000 rpm for 10 min at room temperature, after which the clear supernatant containing genomic DNA was collected.

### DNA precipitation

Sodium acetate stock (3 M) was added to the supernatant obtained after protein precipitation so that the working concentration of sodium acetate was 0.3 M in solution. This was followed by the addition of 200 μl of ice cold ethanol. The solution was vortexed for 5 s and allowed to stand for 4 h at − 30 °C for the DNA to precipitate after which the samples were centrifuged at 15000 g for 1 h at 4 °C. The supernatant was discarded and the pellet was washed twice with 75% ice cold ethanol. Each wash step was followed by centrifugation at 15000 rpm for 10 min at 4 °C. This was followed by a final wash with 100% ice cold isopropanol, after which the wash solution was removed and the pellet was left to air dry in a laminar flow hood. After 7 to 10 min of drying, 10 μl of deionized water or 1X TE was added to the pellet and it was incubated at 55 °C for 10 min to facilitate solubilisation. The quality and quantity of DNA were measured using a NanoDrop.

### PCR reaction

We used four different primer pairs, three pairs were targeted against avian sex chromosomes Z1 (forward: GTGTAGTCCGCTGCTTTTGG), Z2 (reverse: GTTCGTGGTCTTCCACGTTT), W1(forward: GGGTTTTGACTGACTAACTGAT), W2 (reverse: GTTCAAAGCTACATGAATAAACA) [9], P2 (TCTGCATCGCTAAATCCTTT), P8 (CTCCCAAGGATGAGRAAYTG) [8], and one pair was used for amplification of the δ-Opioid Receptor gene, δ-OR (forward: TTCAACCTGGCTCTGGCTGATG), δ-OR (reverse: GTCAATAGAGAGCACAACCTTGC) in extracted DNA samples from males and females of both species.

**Fig. 1:**
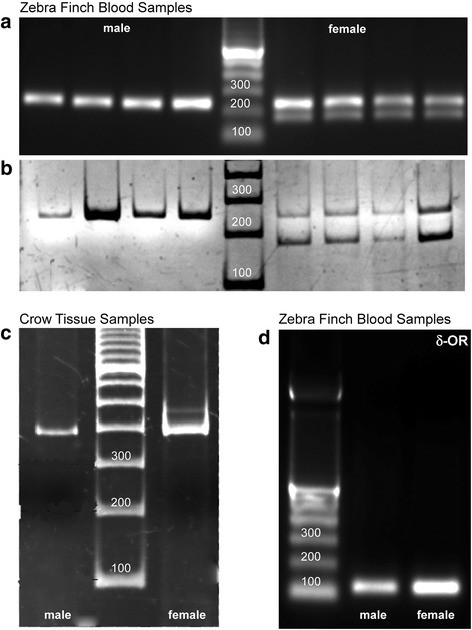
Gel electrophoresis following PCR for zebra finch blood samples. Single bands were obtained for a male at 242 bp and double bands at 242 and 179 bp for a female performed using (**a**) 2% agarose gel and (**b**) 8% polyacrylamide gel respectively; each lane beginning from the marker to the edge of the gel represents double, single, half and one fourth Whatman filter paper disc respectively for both male and female sample. Separate sets of samples were run on agarose and polyacrylamide gels respectively, to demonstrate better band separation of the PCR product obtained from female zebra finch genomic DNA sample by using the latter electrophoresis method. **c** PCR products from crow tissue samples showing a single band at 390 bp for ZZ chromosomes of males and double bands at 390 and 460 bp for Z and W chromosomes for females. **d** A single band was obtained for the delta opioid receptor gene at 120 bp for both male and female zebra finches

Amongst different species, male birds possess homomorphic sex chromosomes, that is, they are arranged as ZZ, whereas females are heteromorphic and have a ZW chromosomal arrangement. As a result, the PCR products from zebra finch blood samples appeared as a single band in case of males at 242 bp and for female sex chromosomes at 242 bp and 179 bp using Z1/Z2 and W1/W2 primer pairs (Fig. 1a; agarose and Fig. 1b; polyacrylamide gel, performed on separate sets of blood samples for proper visualization of band separation for the female PCR product). For crow tissue samples, the PCR products appeared as a single band in case of males at 390 bp and double bands in case of females at 390 bp and 460 bp using the P2/P8 primer pair (Fig. 1c). Using zebra finch blood samples for detecting the delta opioid receptor gene, we found a sharp band at 120 bp in zebra finch DNA samples (Fig. 1d). Zebra finch genomic DNA samples were amplified for sex identification using Z1 Z2 and W1 W2 primers beginning with denaturation at 94 °C for 15 min followed by 30 cycles of 94 °C for 30 s, 56 °C for 45 s and 72 °C for 45 s, terminating with a final incubation at 72 °C for 7 min. For P2 P8 primer pairs the amplification was begun with an initial denaturation at 94 °C for 2 min followed by 45 cycles of 94 °C for 30 s, 48 °C for 45 s and 68 °C for 45 s, ending with a final cycle of 68 °C for 10 min.

### Gel Electrophoresis

Gel electrophoresis was performed using 2% agarose gels, but the resolution between Z and W bands for the crow samples was very poor. To improve the band resolution, electrophoresis was repeated using 8% polyacrylamide gels, which were stained with ethidium bromide (Table 1). Images were acquired using a Syngene Gel Doc (G:Box, Syngene; Gene Sys software).

### Statistics

Software from SigmaPlot, Version 12.0 was used to run Mann-Whitney U tests to compare the DNA yield and quality obtained from samples using the protocol published in Soderstrom (2007) [9] and our method.

### Troubleshooting

The following steps are critical for this extraction protocol. Firstly, 5% Chelex solution should be stirred constantly to ensure that it is equally distributed in all the tubes. Secondly, since Chelex beads tend to be sticky, the tip of the micropipette needs to be cut slightly prior to pipetting out the solution with the resin into Eppendorf tubes. Thirdly, the stock solution of ammonium acetate should be stored at 4 °C and used within a month.

Finally, the DNA pellet is occasionally too small to visualize after precipitation. In such cases, the washing and dissolution steps should be carried out as mentioned after which the purity of the DNA should be assessed by measuring its absorbance using a spectrophotometer followed by electrophoresis on agarose gel.

## Results

**Fig. 2:**
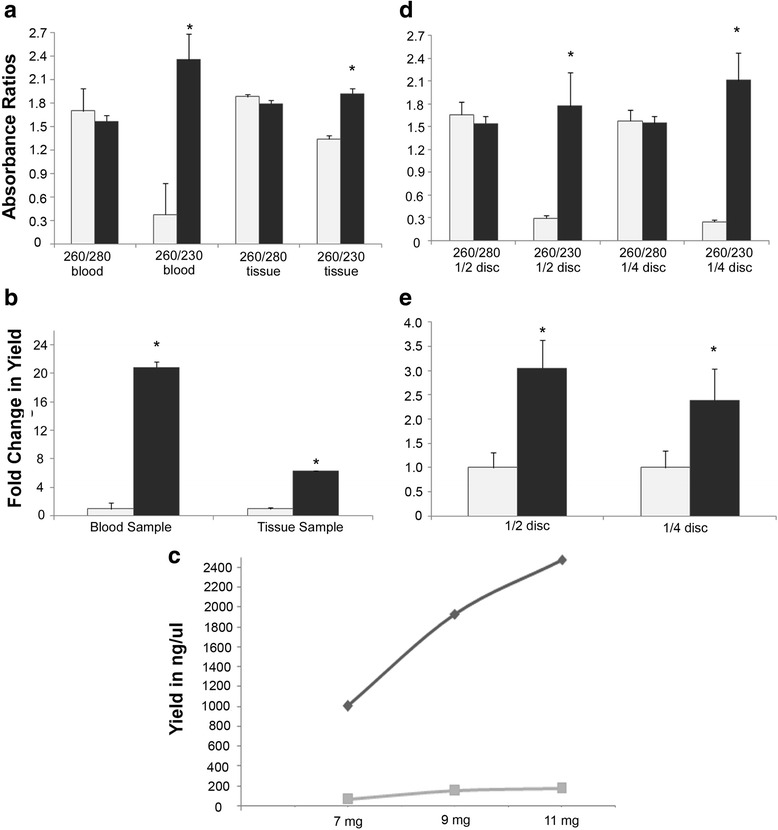
Yield and quality of DNA using the old and modified Chelex protocols. Bar graphs representing median values showing the absorbance ratio (**a**) and yield (**b**) of DNA obtained from blood and tissue samples. Bar graphs in (**d**) represent absorbance ratio and in (**e**) represent yield obtained from half and one fourth of filter paper discs containing the blood sample. White bars represent data obtained by using the old [9] Chelex protocol whereas black bars represent data from the modified Chelex protocol. Error bars represent standard deviations. (**c**) Minimum quantities of tissue samples utilised for DNA extraction are represented by line graphs wherein the dark gray line represents the yield obtained from the modified method whereas light gray line represents the yield obtained from the old Chelex method

We used a modified Chelex DNA isolation protocol, which involved removal of protein debris and purification of DNA. We observed a 6.3-fold improvement [median values of absorbance: older protocol [9]= 0.375, current protocol = 2.370; Mann-Whitney U statistic = 0, *T* = 497, *P* < 0.001] in the ratio of absorbance at 260 nm and 230 nm, whereas there was a 20-fold improvement in the yield of genomic DNA from the blood sample (Mann-Whitney U statistic = 0, T = 497, P < 0.001; median values, old protocol = 13.20, modified protocol = 275.25). However, there was little effect on the absorbance ratio at 260 nm and 280 nm (Mann-Whitney U statistic = 140, *T* = 245, *P* = 0.138; median values, old protocol = 1.70, modified protocol = 1.56; Fig. 2a, b). The Chelex DNA isolation protocol is seldom used to isolate genomic DNA from tissue samples. However, we decided to try our modified version of this protocol on samples of liver tissue and checked the yield before and after DNA precipitation and washing. We observed a 6.3-fold improvement in the yield of genomic DNA from approximately 20 mg of liver tissue (Mann-Whitney U Statistic = 0, *T* = 10, *P* = 0.006, median values before DNA precipitation = 634, median value after DNA precipitation = 3971.3; Fig. 2b) compared to results obtained by the older method [9].

As stated in the Methods section, we wanted to assess the minimum amount of biological sample (blood or liver tissue) required to isolate genomic DNA through our modified protocol. Thus, we halved and quartered the Whatman filter paper disc and subjected it to the modified Chelex method. We observed that both for one fourth and one half of a filter paper disc, there was a significant improvement in the 260/230 nm ratio (1/4th disc, Mann-Whitney U statistic = 0, *T* = 45, *P* < 0.001; 1/2 disc: Mann-Whitney U statistic = 0, T = 45, *P* < 0.001; Fig. 2c). Further, we found that even one fourth of the filter paper disc (approximately 1.25 μl of blood) would yield approximately 93 ng/μl of DNA, which was a 2.4-fold increase compared to the DNA yield from the same amount of blood sample subjected to the old protocol (Mann-Whitney U statistic = 11, *T* = 56, *P* = 0.010, Median = 38.9). Half a Whatman filter paper disc (approximately 2.5 μl of blood) containing the blood sample yielded 207 ng/μl of DNA, which was a 3-fold improvement over the old protocol (Mann-Whitney U Statistic = 2, *T* = 47, *P* < 0.001; Fig. 2D), whereas no difference was observed in the 260/280 nm ratio.

To estimate the minimum measurable amount of tissue sample which may result in a good yield of DNA, different quantities of liver tissue (7 mg, 9 mg and 11 mg) were subjected to the old as well as modified Chelex DNA isolation protocol. We observed that by following our protocol, we could obtain 1000 ng/μl of DNA (260/280 = 1.86, 260/230 = 1.92) from as little as 7 mg of liver tissue **(**Fig. 2e).

**Fig. 3:**
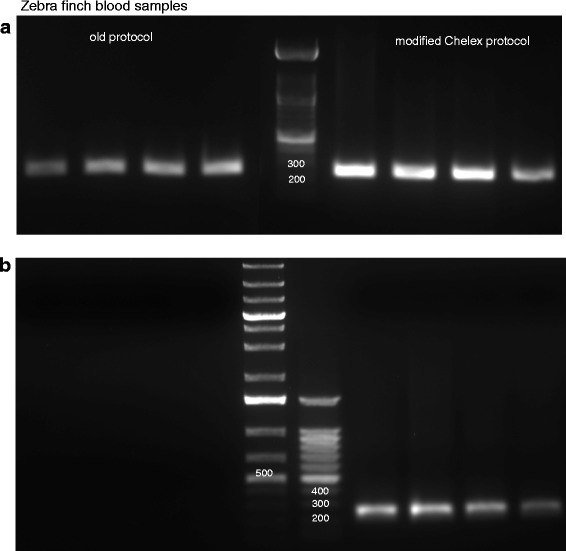
Comparison between PCR products obtained from zebra finch blood samples using the old and modified Chelex method. **a** Clear bands were obtained using the old (left) and modified (right) protocols to analyse the product from Z1/Z2 W1/W2 primer pairs in zebra finch blood samples from adult male birds. The bands represent PCR products obtained from two, one, half and one fourth of a Whatman filter paper disc, beginning at the lane closest to the marker and moving outwards towards the edge of the gel, respectively. **b** The second gel shows no bands obtained by using the old protocol (left), whereas clear bands were obtained by using the modified protocol (right). Band representation remains the same

For comparing the DNA quality obtained using the old and new methods, we compared PCR products of blood samples (of two male zebra finches, shown in Figs. 3a, b) obtained from two, one, half and one-fourth of the Whatman filter paper disc using the Z1/Z2 W1/W2 primer pair. As demonstrated in Fig. 3a, bands were obtained by both old and new methods for the first bird. However, for the second male bird, despite repeating the experiments three times, bands were obtained only using the new protocol (Fig. 3b, right). These results demonstrate that the old method failed to provide consistent results, since we only occasionally obtained clear bands of the amplified DNA.

## Discussion

The Chelex method for DNA extraction has served as a boon for researchers and has provided a high yielding DNA isolation protocol with few chances of contamination. Apart from forensic medicine, this method is now utilised in different fields like microbiology and pathology for detecting genetic traces of pathogens [13–19].

We realised that despite having wide applicability, the purification of DNA samples was limited to proteinase K treatment for removal of proteins [20, 21]. Proteinase K is not only expensive but also needs to be inactivated before performing a PCR as it may digest the polymerase enzyme and inhibit the reaction. Further, we found that most of the reports did not involve any step regarding precipitation of DNA [19, 22–25]. This might result in a DNA template for PCR that is impure, unstable and also prone to inhibition.

The modified protocol for DNA extraction using Chelex 100 explained in this paper, involves a systematic approach to first removing the protein and cellular debris, followed by precipitation and purification of DNA. The aim of this method was to provide a protocol for DNA extraction which was less expensive than silica-based or magnetic separation kits and less toxic than organic extraction using phenol chloroform. This protocol also provides researchers with more control over the experiment and makes troubleshooting easier since much of the debris that may interfere with the PCR reaction is already precipitated out. For the first time, we have attempted to establish a relation between the amount of biological sample required and the quantity as well as quality of DNA obtained.

We have demonstrated that our method not only improved the quality of DNA (a 6.3-fold improvement in the 260/230 ratio) but also enhanced the yield by concentrating the DNA in solution (a 20-fold improvement in the yield compared to the old protocol). We have also shown that minimal quantities of blood or liver samples, that is, a fraction of 5 μl of blood taken on a Whatman filter paper disc 8 mm in diameter yield ample genomic DNA for multiple PCR reactions. Our method also works well for tissues and even trace quantities (7 mg of liver tissue) are able to yield 1000 ng/μl of purified DNA. For comparison, 300 μl of blood yields 5–15 μg, whereas 11 mg of liver tissue yields 15–20 μg of DNA using commercially available kits from Promega [Wizard(R) Genomic DNA Purification Kit Technical Manual TM050]. Similarly, 200 μl of blood yields 4–12 μg whereas 25 mg of brain tissue yields 15-30 μg of DNA using the QiaAmp kit (DNeasy Blood and Tissue Kits, Qiagen; https://www.qiagen.com/). We also performed Trizol based DNA extraction and obtained a yield of 127.9 ng/μl (260/280 = 1.7; 260/230 = 1.12) from 14 mg of tissue, when the DNA was resuspended in 200 μl of 8 M NaOH solution. Further, we have demonstrated that extracting DNA from blood samples using the older Chelex-based protocol is inconsistent and occasionally inhibits the PCR reaction. However, our modified method is consistent across all trials of these experiments for different samples.

Many laboratories use mouse tail tissue or blood from the tail vein for genotyping of mutants, a method which is painful, time-consuming and often causes permanent damage to the tail. Our method uses less than half the volume of blood normally withdrawn from the tail for genotyping, and results in a purified DNA sample with a yield for multiple PCR reactions. Similarly, this method may find applications in determining specific mutations that may have resulted in speciation as well as point mutations that may result in disease. Besides its use in forensic medicine, our protocol may be utilized for genotypic screening and sequencing of specific genes, following the detection of SNPs.

## Conclusions

Despite the fact that DNA extraction using Chelex 100 resin is one of the easiest and most inexpensive methods, there remains a persistent inconsistency in the quality and amount of DNA extract obtained. Impurities and oftentimes Chelex beads carried over into the final DNA extract interfere with downstream processing such as the polymerase chain reaction. In order to avoid inhibition of the PCR reaction and to purify the DNA, we included certain modifications in the Chelex 100 DNA extraction protocol. These changes, involving non-toxic and easily available chemicals, ensure the removal of protein and cellular debris and also the purification and precipitation of DNA, which can be then re-suspended as per the experimenters’ requirements.
